# Unilateral biportal endoscopic discectomy via translaminar approach for highly upward-migrated lumbar disc herniation: a technical note and preliminary treatment outcomes

**DOI:** 10.1186/s12891-024-07819-x

**Published:** 2024-09-07

**Authors:** Wein-Chin Chen, Wei-Ting Wang, Jwo-Luen Pao

**Affiliations:** Department of Orthopedic Surgery, Far-Eastern Memorial Hospital, 21, Section 2, Nanya South Road, Banqiao District, New Taipei, 22060 Taiwan

**Keywords:** Discectomy, Lumbar vertebrae, Endoscopes, Technical report, Treatment outcomes

## Abstract

**Study Design:**

A technical note and retrospective case series.

**Objective:**

Highly upward-migrated lumbar disc herniation (LDH) is challenging due to its problematic access and incomplete removal. The most used interlaminar approach may cause extensive bony destruction. We developed a novel translaminar approach using the unilateral portal endoscopic (UBE) technique, emphasizing effective neural decompression, and preserving the facet joint’s integrity.

**Methods:**

This retrospective study included six patients receiving UBE translaminar discectomy for highly upward-migrated LDHs from May 2019 to June 2021. The migrated disc was removed through a small keyhole on the lamina of the cranial vertebra. The treatment results were evaluated by operation time, hospital stays, complications, visual analog scale (VAS), Oswestry Disability Index (ODI), Japanese Orthopaedic Association (JOA) score, and modified MacNab criteria.

**Results:**

The mean pre-operative VAS for back pain (5.0 ± 4.9), VAS for leg pain (9.2 ± 1.0), JOA score (10.7 ± 6.6), and ODI (75.7 ± 25.3) were significantly improved to 0.3 ± 0.5, 1.2 ± 1.5, 27.3 ± 1.8, 5.0 ± 11.3 respectively at the final follow-up. Five patients had excellent, and one patient had good outcomes according to the Modified MacNab criteria. The hospital stay was 2.7 ± 0.5 days. No complication was recorded. The MRI follow-up showed complete disc removal, except for one patient with an asymptomatic residual disc.

**Conclusions:**

UBE translaminar discectomy is a safe and effective minimally invasive procedure for highly upward-migrated LDH with satisfactory treatment outcomes and nearly 100% facet joint preservation.

## Introduction

Lumbar disc herniation (LDH) is a common degenerative spine disorder that induces lower back pain, radicular leg pain, and disability. Herniated disc fragments, once extruded, might migrate into the spinal canal either cranially or caudally. About one-third of herniations are non-migrated, 16% migrated cranially, and 56% caudally [1]. Among them, the highly upward-migrated type is the most challenging for spine surgeons. For disc herniations without migration, the surgeon may access the disc herniation easily through the interlaminar window with a small laminotomy and minimal facet joint violation.

In contrast, for a highly upward-migrated disc herniation to the pedicle level of the cranial vertebra, a much larger laminotomy and partial facetectomy are unavoidable to overcome the anatomic barrier [2]. Extensive removal of bony structures may lead to untoward segmental instability [3, 4]. MacNab used “hidden zone” to describe this unique type of disc herniation and pointed out the difficulties in surgical exposure of this region [3]. 

To minimize the extensive bony destruction, Di Lorenzo et al. described a microscopic translaminar approach to access the hidden zone by drilling a hole in the cranial lamina and removing the herniated disc through the hole [5]. However, this approach was performed by traditional open technique and might cause excessive soft tissue damage and related late sequels.

Various minimally invasive surgical techniques have been developed for managing degenerative lumbar spinal diseases in the last two decades [6]. Given the constant improvement of instruments and surgical techniques, more surgeons tried to refine their surgical technique from conventional open or mini-open to endoscopic approaches. The unilateral biportal endoscopic (UBE) technique is gaining popularity for its minimal invasiveness, short learning curve, and accessible equipment availability. The UBE technique has been proven to be a safe and effective surgical technique for various degenerative spine disorders, with less blood loss, less postoperative pain, short hospital stays, and early postoperative recovery [7, 8]. 

In this study, we developed the UBE translaminar technique to address the highly upward-migrated lumbar disc herniation. This article aims to describe the surgical technique and report the preliminary clinical outcomes.

## Materials and methods

### Patient selection

**Fig. 1:**
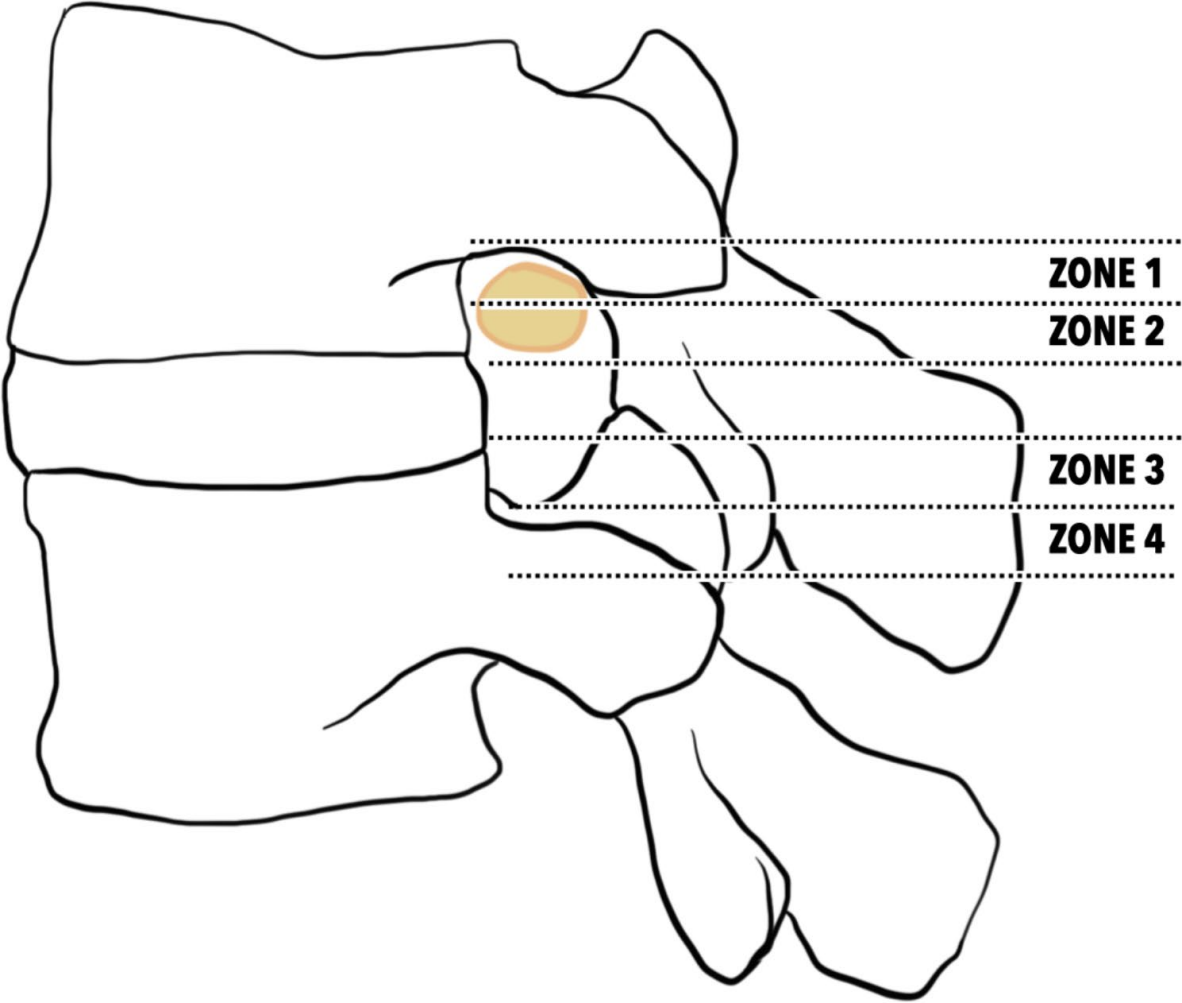
Definition and illustration of the four zones for migrated lumbar disc herniation

The Research Ethics Review Committee of Far Eastern Memorial Hospital (No. 110123-E) approved the study protocols and waived the need for written informed consent due to the study’s retrospective design. From May 2019 to June 2021, 6 patients who underwent a unilateral biportal endoscopic (UBE) discectomy via translaminar approach for highly upward-migrated lumbar disc herniations (LDH) were enrolled in this retrospective study. The indications for surgery were: (1) Radicular leg pain or neurological symptoms/ signs induced by lumbar disc herniation at a single level. (2) Persistent symptoms after conservative management with medical treatment or physical therapy for at least six weeks. (3) Correlated magnetic resonance imaging (MRI) findings to prove the offending pathology. (4) Highly upward-migrated disc herniation in the “hidden zone” or anatomical zone I, by the definition of Lee’s classification (Fig. 1) [9]. Patients with significant segmental instability or had a history of spine surgery were excluded from this study.

### Evaluation of clinical data and outcomes

All operations were performed by a single surgeon in a single medical center. The patient’s demographic data, clinical symptoms and signs, operation time, amount of estimated blood loss, days of hospitalization, follow-up period, and complications were retrospectively reviewed from the medical records. All patients had pre-operative X-rays and MRIs for the lumbar spine. Follow-up MRI was performed three months after surgery. Treatment outcomes were evaluated using the visual analog scale (VAS) for lower leg pain and back pain, the Oswestry Disability Index (ODI) for disability, the Japanese Orthopedic Association (JOA) scores for functional recovery, and the modified MacNab criteria for overall outcomes.

### Surgical technique

The settings and precautions for unilateral biportal endoscopic surgery were previously described [10]. 

After induction of general anesthesia, the patient is placed in a prone position on the radiolucent Relton-Hall frame. The lumbosacral region is disinfected and draped as sterile as usual. Because continuous normal saline irrigation is required when performing the UBE surgery, it is critical to ensure a waterproof draping to prevent soaking and resultant hypothermia. When positioning the patient, ensuring enough free space for passage and adjustment of the fluoroscope is essential to obtain clear anteroposterior and lateral views of the interested intervertebral discs is essential. Instead of tilting the fluoroscope, we can tilt the surgical table to make the disc vertical to the ground. The alternative way is more ergonomic and comfortable for the operating surgeon.

**Fig. 2:**
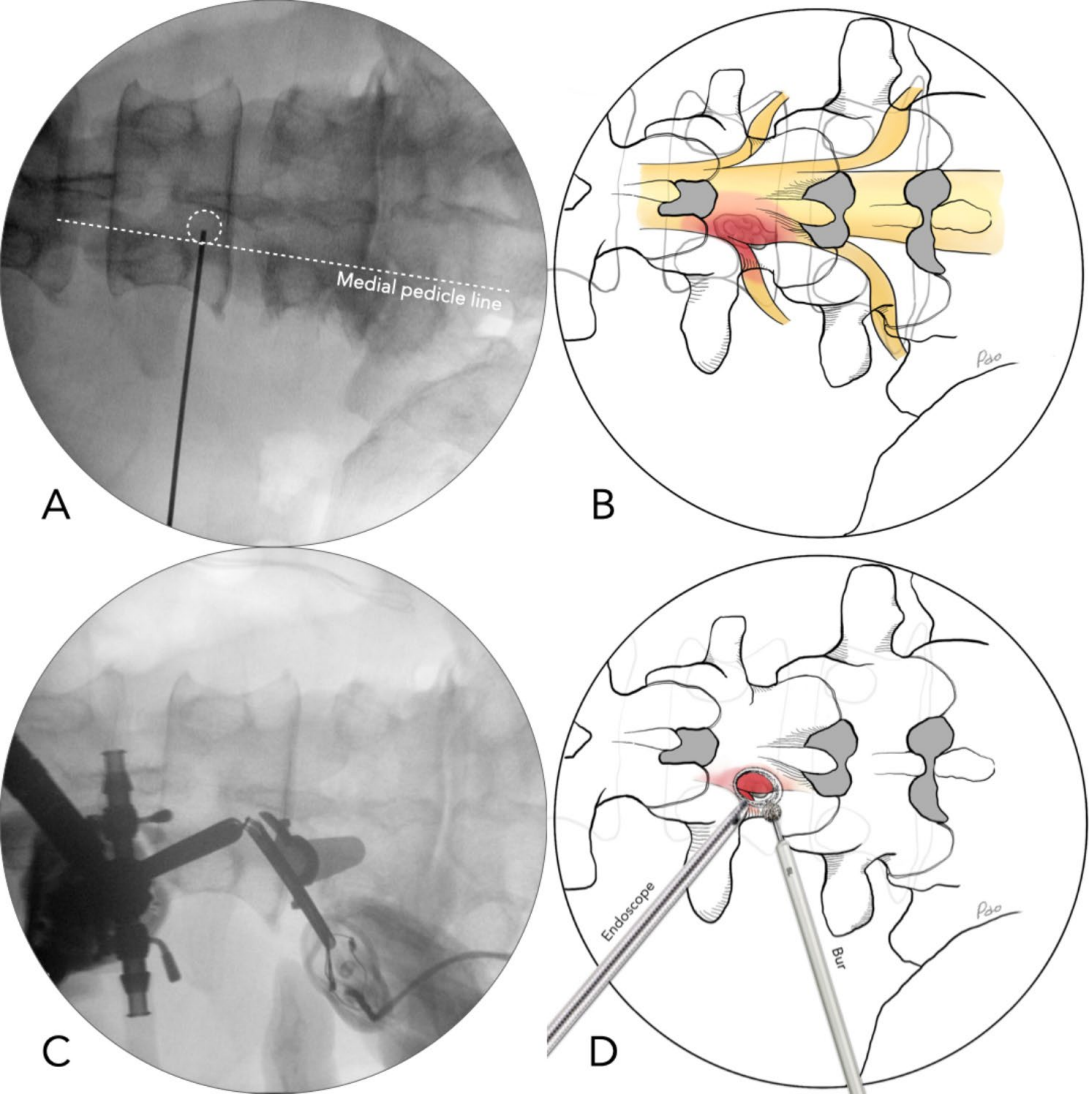
(**A**) Intra-operative fluoroscopic image in the anteroposterior projection. The circle indicates the initial target point at the ipsilateral side of the cranial lamina. (**B**) Schematic illustration for highly upward-migrated lumbar disc herniation located in the hidden zone. (**C**, **D**) An intra-operative fluoroscopic image and schematic illustration show the triangulation formed by the target point, the endoscope, and the surgical instruments

After proper positioning and draping, we use the biplanar fluoroscope and Kirschner wires to localize and mark the interested disc level, the initial target point, and the skin incisions (Fig. 2AB). The initial target point is determined pre-operatively by studying the MRI for surgical planning. Unlike the interlaminar approach, the target point for the translaminar approach is on the lamina instead of at the spinolaminar junction. The two skin incisions are along the medial pedicle line, separated by about 2 ∼ 3 cm, depending on the patient’s body habitus.

We perform the translaminar approach from the ipsilateral side of the lesion. Take the left-side approach, for example. The cranial portal is the viewing portal for the endoscope and saline inflow; the caudal portal is the working portal for the surgical instruments and saline outflow. The skin incision is about 5 mm for the viewing portal and 10 mm for the working portal. We prefer transverse incisions for the skin and the underlying deep fascia for cosmesis and better saline outflow. We use a blunt dilator to penetrate the paraspinal muscles and gently detach them from the lamina.

Triangulating the scope and instrument tips upon the target point is the initial step of each UBE procedure (Fig. 2C). We use a 30-degree 4-mm arthroscope for the translaminar approach. We use the radiofrequency wand (ArthroCare, Austin, Texas, USA) to clean the soft tissue on the lamina and coagulate the bleeders from soft tissues. Fluent saline inflow and outflow are essential to maintain a clear surgical field because they wash away the background oozing and grind bone debris while drilling. A fluent outflow also minimized the extravasation of the saline into the paraspinal muscles.

**Fig. 3:**
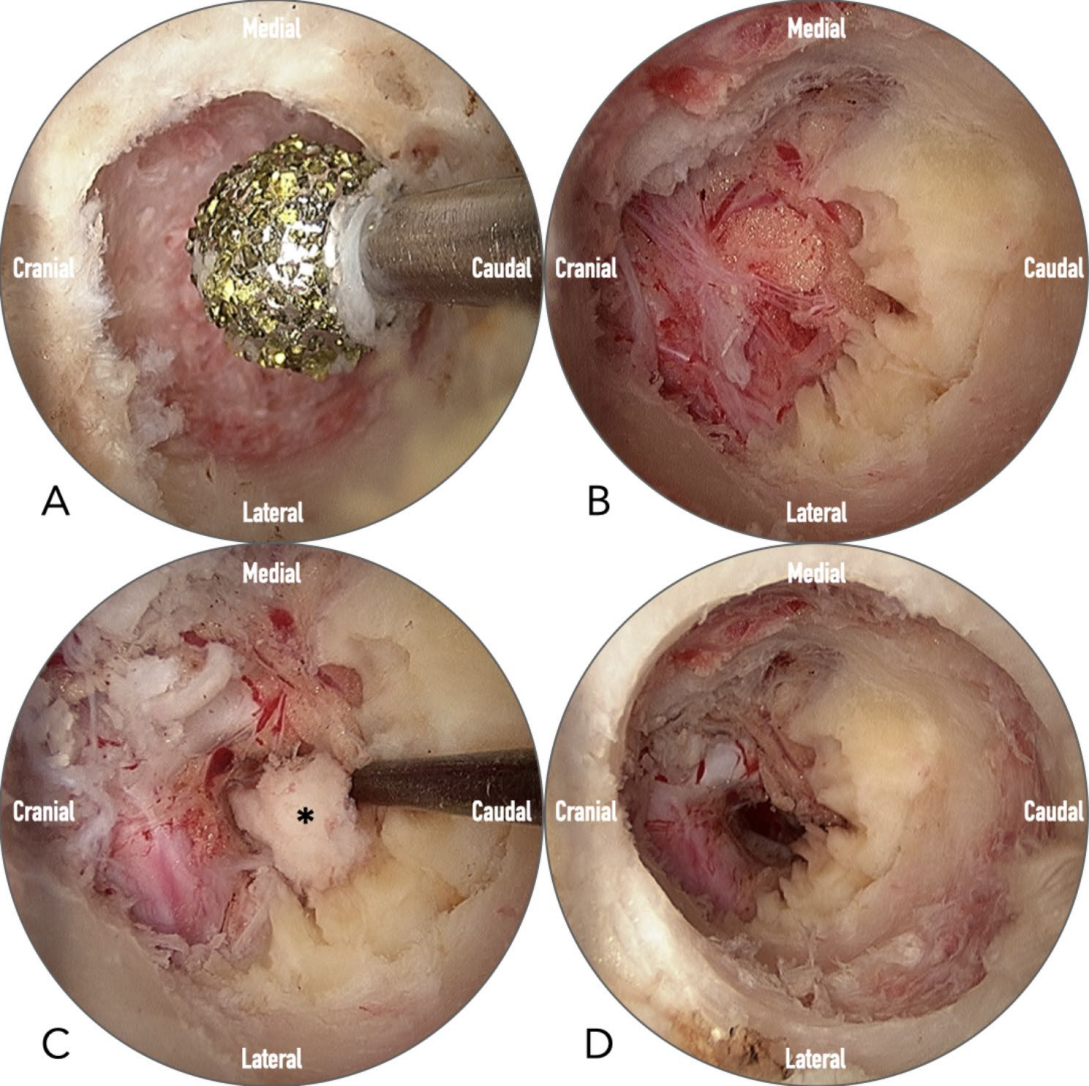
Intra-operative endoscopic images illustrate the processes of translaminar discectomy. (**A**) Creating the crater using a 4 mm high-speed diamond burr. (**B**) Removing the cranial origin of ligamentum flavum to expose the axilla area of the nerve root. (**C**) Probing the herniated disc (asterisk). (**D**) The ruptured disc was removed, and the dura and nerve root were freely mobile with no violation of the facet joint

Before drilling, the precise localization must be re-confirmed under the fluoroscope. Once the starting point is confirmed, the lamina is gently drilled through using a 4 mm oval coarse diamond burr till a sudden giving-way feedback from the tip of the drill (Primado 2, NSK, Fukushima, Japan) (Fig. 2D). The fluoroscope is used frequently to determine the location of the burr hole. Under the endoscope, the size of the burr hole or laminotomy can be evaluated using the burr tip (4 mm in diameter) as a reference (Fig. 3A). A laminotomy about 8 mm in diameter is usually big enough for discectomy. However, further enlargement might be necessary, depending on the location of the ruptured disc and the accessibility of the surgical instruments. The cranial attachment of the ligamentum flavum was identified after drilling—only the cranial part of the ligamentum flavum needed to be removed to expose the neural tissues.

The ruptured disc is usually located in the axilla area or upward migrated underneath the affected nerve root (e.g., the L4 root was usually the affected root in L4-5 high upward migrated disc herniation). The ruptured disc may induce a severe inflammatory reaction, which leads to adhesion and engorged vessels around the neural tissues. Bleeding from the engorged vessels is a severe problem and must be controlled using meticulous hemostasis techniques. To avoid a dura tear or nerve root injury, we use a small nerve hook to release the adhesion and a blunt neural dissector to mobilize the dura and the nerve root. Most of the time, the ruptured disc would pop out, and we can remove the main fragment using small pituitary forceps. (Fig. 3B-C). It is crucial to probe the dead space to ensure no ruptured disc fragments are left behind (Fig. 3D). We will try to find the annular defect and perform thermal annuloplasty using the radiofrequency wand to lower the risk of recurrence. The closed suction drain tube is optional once meticulous hemostasis is achieved.

The numeric data, including VAS for back and leg pain, ODI, and JOA scores between preoperative and final follow-up, was analyzed using Student’s t-test. A *p*-value of less than 0.05 was considered statistically significant.

## Results

In total, six patients with highly upward-migrated LDH who received UBE translaminar discectomy were included in our study. All patients complained of sudden onset of lower back pain with radiation into the lower leg of the affected side while performing daily activities or after lifting a heavy object. They came to our clinic for help because there was no significant improvement with conservative management, including physical therapy, oral medications, or local injections for at least six weeks. Our study included four men and two women with a mean age of 58.5 ± 8.2 years (49–68 years). The involved disc levels were L3-4 in 1 patient, L4-5 in 4 patients, and L5-S in 1 patient. The average operation time was 56.7 ± 8.2 min (40–60 min). Length of hospital stays was 2.7 ± 0.5 days (2–3 days). The intra-operative blood loss was too little to be measured because it was diluted in a vast amount of normal saline, estimated to be around 5mL. (Table 1).

**Table 1:** Summary of demographic data

Gender	Male	4
	Female	2
Age (years)		58.5 ± 8.2
Level	L3-4	1
	L4-5	4
	L5-S	1
Operation time (minutes)		56.7 ± 8.2
Hospital stays (days)		2.7 ± 0.5

**Fig. 4:**
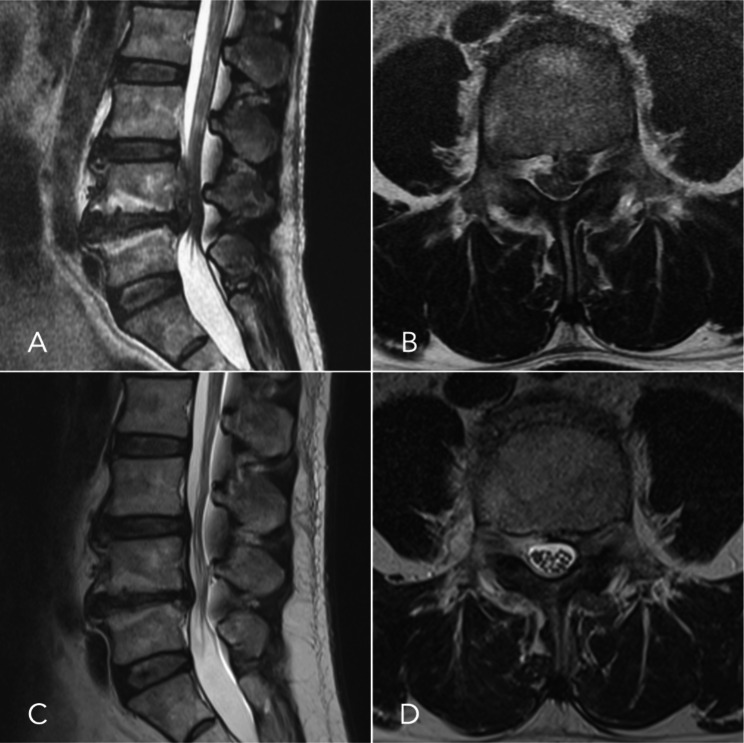
(**A**, **B**) Pre-operative MRI revealed a highly upward-migrated disc herniation extending beyond zone 1 with thecal sac compression at L4–5 level. (**C**, **D**) Post-operative MRI showed that the ruptured disc was removed entirely with re-expansion of the thecal sac and no soft tissue injury

All the patients had significant improvement in back pain, leg pain, and neurological symptoms immediately after surgery. The average follow-up period was 17.0 ± 9.8 months (6–28 months). Compared with the pre-operative conditions, the VAS for back pain was improved from 5.0 ± 4.9 to 0.3 ± 0.5 at the final follow-up. The VAS for leg pain was improved from 9.2 ± 1.0 to 1.2 ± 1.5. The JOA scores were improved from 10.7 ± 6.6 to 27.3 ± 1.8. The ODI was improved from 75.7 ± 25.3 to 5.0 ± 11.3. According to the modified MacNab criteria, the treatment outcomes were excellent in 5 patients and good in 1 patient (Table 2). On follow-up X-rays, we did not observe significant disc space narrowing or segmental instability. There were no complications or recurrence of disc herniation at the final follow-up. All post-operative MRIs showed complete removal of the ruptured disc with no facet joint injury and almost no soft tissue damage, except a small residual disc was noted in 1 patient with no clinical symptoms (Fig. 4).

**Table 2:** Summary of treatment outcomes

	Preoperative	Final follow-up	*p*-value
VAS back pain	5.0 ± 4.9	0.3 ± 0.5	0.04
VAS leg pain	9.2 ± 1.0	1.2 ± 1.5	< 0.01
ODI	75.7 ± 25.3	5.0 ± 11.3	< 0.01
JOA score	10.7 ± 6.6	27.3 ± 1.8	< 0.01
MacNab outcome	Excellent	5 (83.3%)	
	Good	1 (16.7)	
	Fair	0	
	Poor	0	

VAS = visual analog scale; ODI = Oswestry disability index; JOA = Japanese Orthopedic Association

## Discussion

For patients with lumbar disc herniations, a discectomy was indicated for relieving low back pain and sciatica, especially in those who did not have significant improvement with conservative treatment for 6 weeks [11]. The well-known randomized controlled study from Spine Patient Outcomes Research Trial (SPORT) demonstrated that the patients who underwent surgical intervention showed significantly greater improvement in pain, function, satisfaction, and self-rated progress compared to patients treated non-operatively after eight years of follow-up [12]. The Maine Lumbar Spine Study (MLSS) also concluded that patients who underwent surgical intervention had better leg pain relief and greater back-related functional status [13]. 

Microdiscectomy using Love’s method had been the standard surgical procedure for lumbar disc herniation for decades. To minimize the soft tissue damage caused by surgical exposure, Kambin and Gellman first proposed the concept of percutaneous lateral discectomy in 1983. They inspired the idea of the minimally invasive spine surgery [14]. Various minimally invasive techniques, including microendoscopic technique, percutaneous endoscopic technique, and biportal endoscopic technique, were developed to overcome the limitations of minor surgical wounds yet provide effective decompression of the neural tissues [6, 15–17]. The patients benefit from minor surgical wounds, less soft tissue damage, less post-operative pain, faster recovery, and better treatment results than the traditional open or microscopic techniques [7, 8, 18]. However, for the highly upward-migrated LDH, Love’s method may lead to extensive lamina and facet joint destruction, which raises concerns about subsequent segmental instability.

Percutaneous endoscopic lumbar discectomy has been successfully used to treat various types of lumbar disc herniation via the transforaminal approach [19]. With the advancement of endoscopic technology, the herniated disc can be removed through a surgical wound of about 8 mm with minimal bony destruction [20, 21]. However, for highly upward-migrated LDH, the failure rate was as high as 15.7% [22]. In a retrospective study that enrolled 10,228 patients, 283 out of 436 failure cases requiring re-operation were attributed to incomplete removal of the herniated discs. Of the unsuccessful cases, 24.7% were found to have migrated herniated discs [23]. 

The translaminar approach was proposed to tackle the migrated disc in the hidden zone [5]. Instead of widening the interlaminar window to access the highly upward-migrated disc, the translaminar approach creates a crater on the cranial lamina overlying the migrated disc. Only partial excision of the ligamentum flavum is necessary to expose the neural structures. The disc material can be identified underneath the nerve root in the axilla area. The advantages of this approach are to minimize the extent of laminotomy, preserving most parts of the ligamentum flavum, leaving the interlaminar window undisturbed, thus minimizing postoperative epidural scarring and maintaining the integrity of the facet joint. Although the translaminar approach can be done by traditional open technique [5], microscopic technique [4, 24], or microendoscopic technique [25], soft tissue damage is the major drawback of these techniques. It can also be done by percutaneous endoscopic technique [2, 26]. However, the steep learning curve and expansive endoscopic equipment are the primary concerns.

The unilateral biportal endoscopic (UBE) technique has been developed since 2003 as a minimally invasive surgical technique and has been applied to various spine disorders successfully [8, 27–32]. The surgeon operates with the endoscope in his left hand and the surgical instrument in his right hand. Compared with the percutaneous uniportal endoscopic technique, the UBE technique is more ergonomic and makes it easier to overcome the learning curve [33–35]. The equipment is already available in every hospital that can perform orthopedic arthroscopic surgery, and it is much cheaper. Bleeding control is easy with the radiofrequency wand by controlling the hydrostatic pressure of normal saline and using the bone wax to seal the oozing from the bone. Using the 30-degree endoscope with a high-resolution camera system, we can get a wide surgical field in a tiny space and good differentiation between different tissues by magnification. Therefore, we can perform delicate manipulation of the neural tissue without a dura tear or nerve injury.

Highly upward-migrated disc herniation in the hidden zone might be challenging, but achieving adequate decompression with the UBE translaminar approach is not difficult. In our study, 83% of patients had excellent outcomes rated by the modified MacNab criteria at a mean follow-up of 17 months without complications or recurrences. Our results also showed that the patients had significant pain reduction on the next day of operation and significant improvement in the JOA score and ODI at the final follow-up, indicating the quick recovery and long-lasting treatment effect achieved with the UBE translaminar approach.

Incomplete removal of the migrated disc is an essential cause of failure for highly upward-migrated LDH. Xu et al. reported that around 65% of unsuccessful discectomies with percutaneous endoscopic technique were attributed to incomplete removal of discs, of which 70% were classified as distant migrated discs [20]. In our MRI follow-up, all the ruptured discs were completely removed except a small residual disc was observed in only one patient with no clinical symptoms. This result supports that the UBE translaminar approach is effective for highly upward-migrated lumbar disc herniation.

Although the UBE technique is relatively easy to learn, the learning curve is still an essential concern for beginner surgeons. The learning curve was estimated to be 30 cases for spine surgeons without experience in endoscopic spine surgery and 10 to 15 cases for surgeons involved in microendoscopic or percutaneous endoscopic procedures previously [33]. Mentorship from an experienced surgeon is highly recommended to avoid possible complications.

Our study’s limitations include strict patient selection, a small number of cases, short-term follow-up, and a lack of a control group. Large-scale studies with more patients, a long-term follow-up, and a comparative study with other surgical techniques are necessary to prove the efficacy and safety of the UBE translaminar approach for highly upward-migrated LDH.

## Conclusion

UBE translaminar approach can be a feasible, safe, and effective surgical technique to address highly upward-migrated LDH. The treatment outcomes are encouraging, with minimal soft tissue damage, nearly 100% preservation of the facet joints, good pain relief, and good functional recovery. Furthermore, this approach’s relatively friendly learning curve offers the surgeon an easier way to start.

## Data Availability

The datasets used and/or analysed during the current study are available from the corresponding author upon reasonable request.
